# Primary cardiac osteosarcoma in a 42-year-old woman

**DOI:** 10.1186/1749-8090-5-120

**Published:** 2010-11-27

**Authors:** Honghe Luo, Yiyan Lei, Chunhua Su, Lie Cai, Tao Wang, Jianyong Zou, Zhenguang Chen

**Affiliations:** 1 Department of Thoracic Surgery, The First Affiliated Hospital, Sun Yat-sen University, Guangzhou (510080), Guangdong, People's Republic of China; 2 Department of Rehabilitation, The First Affiliated Hospital, Sun Yat-sen University, Guangzhou (510080), Guangdong, People's Republic of China; 3 Center for Stem Cell Biology and Tissue Engineering, Sun Yat-sen University, Key Laboratory for Stem Cells and Tissue Engineering, Ministry of Education, Guangzhou (510080), Guangdong, People's Republic of China

## Abstract

We describe here a 42-year-old woman who was admitted to hospital with a pedunculated mass in her left atrium. She was diagnosed with a primary cardiac osteosarcoma with special immunohistochemical characteristics. Echocardiography and computed tomography can be used to differentiate cardiac osteosarcomas from routine intracardiac tumors. The patient was treated by surgical removal of the mass. Two years later, she has shown no evidence of disease recurrence. We discuss primary osteosarcomas in the cardiac cavity and their management.

## Introduction

Although osteosarcoma is a common tumor of the skeletal system, primary cardiac osteosarcoma is an extremely rare malignant disease with nonspecific symptoms, making early diagnosis a challenge. We describe here a 42-year-old woman with a primary cardiac osteosarcoma, which was surgically removed by cardiopulmonary bypass. Two years later, she has shown no evidence of tumor recurrence.

## Case report

**Figure 1:**
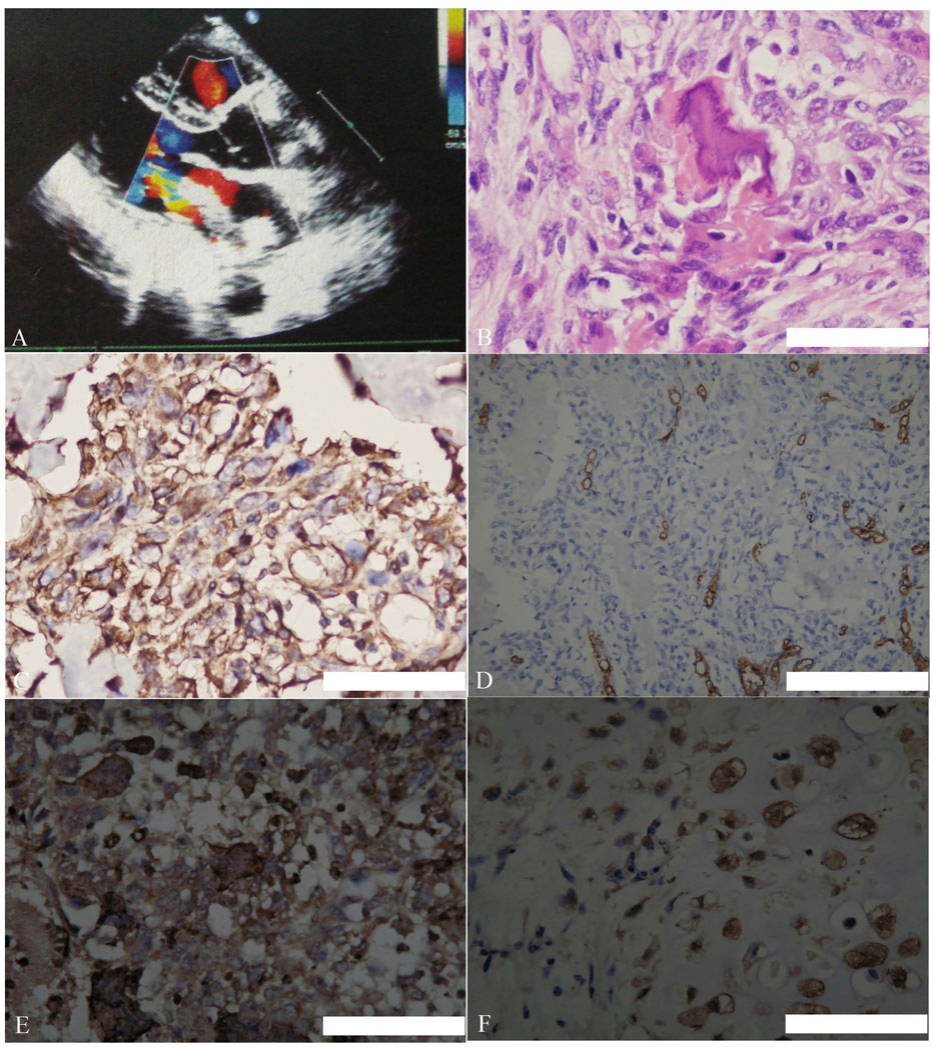
**Characteristic of the primary cardiac osteosarcoma in our patient**. (**A**) Echocardiography results, showing a mass in the left atrium with accelerated color flow across the mass, suggesting a hemodynamically significant obstruction. The mitral valve area was 2.5 cm^2^. (**B**) Histopathologic examination, showing that, microscopically, the tumor was composed of a uniform population of large atypical cells with prominent nucleoli and an osteogenic sarcomatous element. Original magnification ×400; (**C-F**) Immunohistochemical results, showing that the tumor was strongly stained with antibodies to vimentin (C) and CD68 (E), weakly stained with antibodies to CD34 staining (D), and completely negative for S100 (**F**). Original magnification ×400. Bar, 100 μm.

A 42-year-old woman was admitted to our hospital complaining of chest pain, shortness of breath and weight loss. Physical examination revealed an extra systolic murmur at the cardiac apex, with NYHA stage III. An electrocardiogram revealed sinus bradycardia, and echocardiography showed a pedunculated mass in her left atrium with weak aortic and mitral valve insufficiency, similar to myxoma (Figure 1). Computed tomography revealed a mass, 65 × 20 × 20 mm in size and attached to the posterior wall of the left atrium, without calcification or pericardial effusion. The patient was diagnosed with a primary cardiac tumor and was referred for surgical removal of the mass. During surgery, a tumor measuring 50 × 20 × 20 mm was found, with a stalk attached to the posterior wall of the left atrium and near the orifice of the left pulmonary vein. The mass was removed and a partial endocardiectomy was performed. Pathological examination of the tumor showed that the malignant cells were irregularly osteoid without polygonal to stellate shapes. The tumor cells were strongly stained with antibodies to the osteoclast marker CD68 and vimentin, but were weakly stained with antibodies to CK, EMA, S-100, and CD34 (Figure 1). Based on these histological and immunohistochemical findings, the final diagnosis was primary cardiac osteosarcoma [1,2]. At present, 2 years after surgical removal of the tumor, the patient remains healthy with no evidence of tumor recurrence.

## Discussion

Most primary cardiac tumors are myxomas, and only a very small proportion of these cardiac tumors (< 0.28%) are malignant [3]. Only a few isolated cases of primary cardiac osteosarcoma have been reported, making the etiology of these tumors unclear [1-5]. To our knowledge, therefore, primary cardiac osteosarcomas are rare and difficult to diagnose.

The symptoms of primary cardiac osteosarcoma have been described as protean, with obstruction and heart failure being the primary manifestations [1,3]. On echocardiography, cardiac osteosarcomas often show asymmetrical internal echoes, and computed tomography has shown the calcification of cardiac osteosarcomas. Certain features (e.g., a broad base of attachment or origin at a site other than the atrial septum) help differentiate these tumors from left atrial myxomas [6]. However, the tumor in our patient presented as a soft symmetrical parenchymal tumor, the presence of calcification did not seem useful in differentiating atrial osteosarcoma from myxoma.

Cardiopulmonary bypass is essential for removing the primary cardiac osteosarcoma. We chose a right angle type superior vena cava tube to avoid crushing the tumor in our patient. The mass was removed, along with at least 5 mm of the surrounding endocardium. Because of the risks of tumor fragmentation and embolization, vigorous manipulation should be avoided during surgical treatment.

In brief, we have shown that, although rare, primary cardiac osteosarcoma should be included in the differential diagnosis of patients with neoplasms in the cardiac cavity.

## Consent

Written informed consent was obtained from the patient for publication of this case report and accompanying images. A copy of the written consent is available for review by the Editor-in-Chief of this journal.

## Competing interests

The authors declare that they have no competing interests.

## Authors' contributions

HL and ZC conceived the study and drafted the manuscript. YL, CS and LC managed the histopathological analysis of tumor sample and participated in the manuscript preparation. TW participated in the figure preparation. All authors read and approved the final manuscript.
